# Intraoperative cone-beam computed tomography for catheter placement verification in pediatric hydrocephalus: technical note

**DOI:** 10.1007/s00381-024-06592-5

**Published:** 2024-09-04

**Authors:** Matthias Krause, Jasmina Lagumdzija, Simon Enzinger, Jörn Wittig, Alexander Gaggl, Roman P. Metzger, Christoph J. Griessenauer

**Affiliations:** 1 Department for Neurosurgery, Paracelsus Medical University, Christian Doppler Klinik, Müllner Hauptstrasse 48, 5020 Salzburg, Austria; 2 Department of Pediatric Surgery, Paracelsus Medical University, University Hospital, Salzburg, Austria; 3 Doctoral Degree Program in Medical Science, Paracelsus Medical University, Strubergasse 21, 5020 Salzburg, Austria; 4 Department of Oral and Maxillofacial Surgery, Paracelsus Medical University, University Hospital, Salzburg, Austria; 5 Department of Neurosurgery, University Hospital Leipzig, Liebigstrasse 20a, 04103 Leipzig, Germany

**Keywords:** Intraoperative navigation, Cone-beam computed tomography, Pseudotumor cerebri, Shunt placement, Pediatric neurosurgery

## Abstract

Ventriculoperitoneal (VP) shunt placement, essential for managing hydrocephalus, often risks catheter malpositioning, especially in patients with small ventricles. We present a novel technique combining neuronavigation with intraoperative cone-beam computed tomography using the BrainLab system and Loop-X mobile imaging unit. This approach enables real-time verification of catheter placement by integrating preoperative MRI data with intraoperative CT imaging. In a 12-year-old boy with therapy-refractory idiopathic intracranial hypertension, neuronavigation was guided by the BrainLab Skull Fix and Cushing canula, ensuring precise catheter insertion into the right frontal horn. Post-placement, Loop-X facilitated immediate verification of the catheter’s trajectory and positioning, corroborated by postoperative MRI. This technique demonstrated high precision and minimized radiation exposure, emphasizing its utility in reducing revision rates due to suboptimal catheter placement.

## Introduction

Ventriculoperitoneal (VP) shunt placement is a common procedure that harbors significant risk of malpositioning of the ventricular catheter [1–6]. Suboptimal position of the catheter tip may have a negative impact on short- and long-term revision-free functioning of the shunt [1, 2, 4, 5]. The risk of suboptimal catheter placement is highest in patients with small ventricles, e.g., slit ventricle syndrome of idiopathic intracranial hypertension (IIH, pseudotumor cerebri). Real-time imaging for catheter placement is desirable to verify the correct positioning of the catheter tip intraoperatively [2, 3, 8, 9]. Various authors have described the use of neuronavigation with preoperative MRI set or stereotactic techniques [2, 5, 7]. Intraoperatively, ultrasound with burr-hole probes may be used [2, 8, 9]. However, the technique is limited by reduced imaging quality and the need to enlarge the burr-hole to simultaneously fit in the probe and catheter. Alternatively, commercially available navigation stylets for the use with ventricular catheters proved to be a useful tool for real-time navigation [10]. However, the sole use of neuronavigation lacks the option of immediate intraoperative verification of the tip positioning. Hence, it is not possible to rule out deviations caused by inaccuracy of the navigation system.

Here, we describe our technique of neuronavigation-assisted ventricular catheter placement combined with an intraoperative imaging via cone-beam computed tomography to verify catheter position in pediatric neurosurgery in a first patient.

## Technical note

The operating room utilized by oro-maxillo-facial and neurosurgery is equipped with a BrainLab neuronavigation system (BrainLab, Munich, Germany) combined with the Loop-X mobile imaging unit (medPhoton, Salzburg, Austria). The intraoperative cone-beam computed tomography scanner can be used to automatically co-register real-time CT data with preexisting MRI data during the surgical procedure. After scan acquisition, the CT scan will be automatically imported into the BrainLab. The acquisition time for a CT scan is about 5 min including additional drape coverage of the surgical field and moving the scanner to and from the operating table.

### Case example

**Fig. 1:**
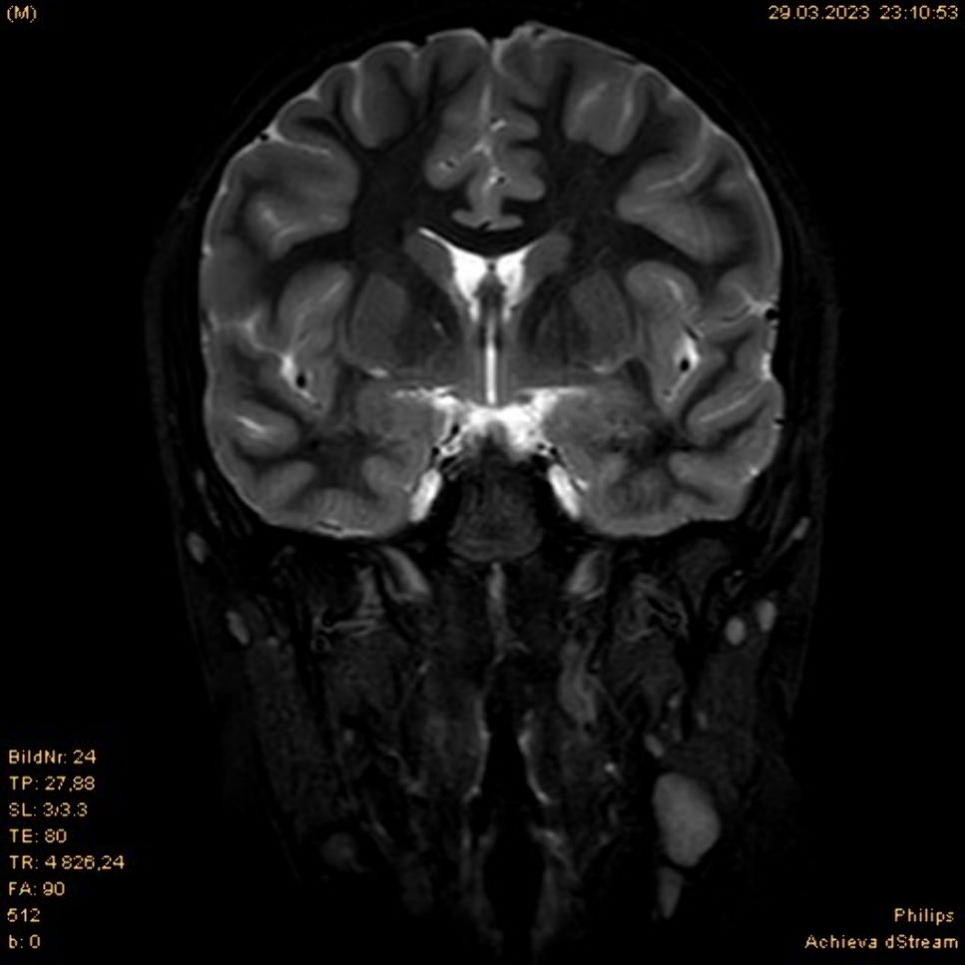
Preoperative cranial MRI in coronal orientation and T2-weighted sequence. Due to the underlying pathology of idiopathic intracranial hypertension, supratentorial ventricular system is very narrow

A 12-year-old boy suffered from therapy-refractory idiopathic intracranial hypertension with progressive visual impairment. A VP shunt insertion was necessary with preoperative MRI verifying very narrow ventricles (Fig. 1). Thus, neuronavigation-assistance was warranted for correct placement of the ventricular catheter. Neuronavigation was enabled using the BrainLab Skull Fix referencing system applied to the cranium at a left frontal position with 5 mm screw to enable free intraoperative movement of the head for passing the catheter to the abdomen.

**Fig. 2:**
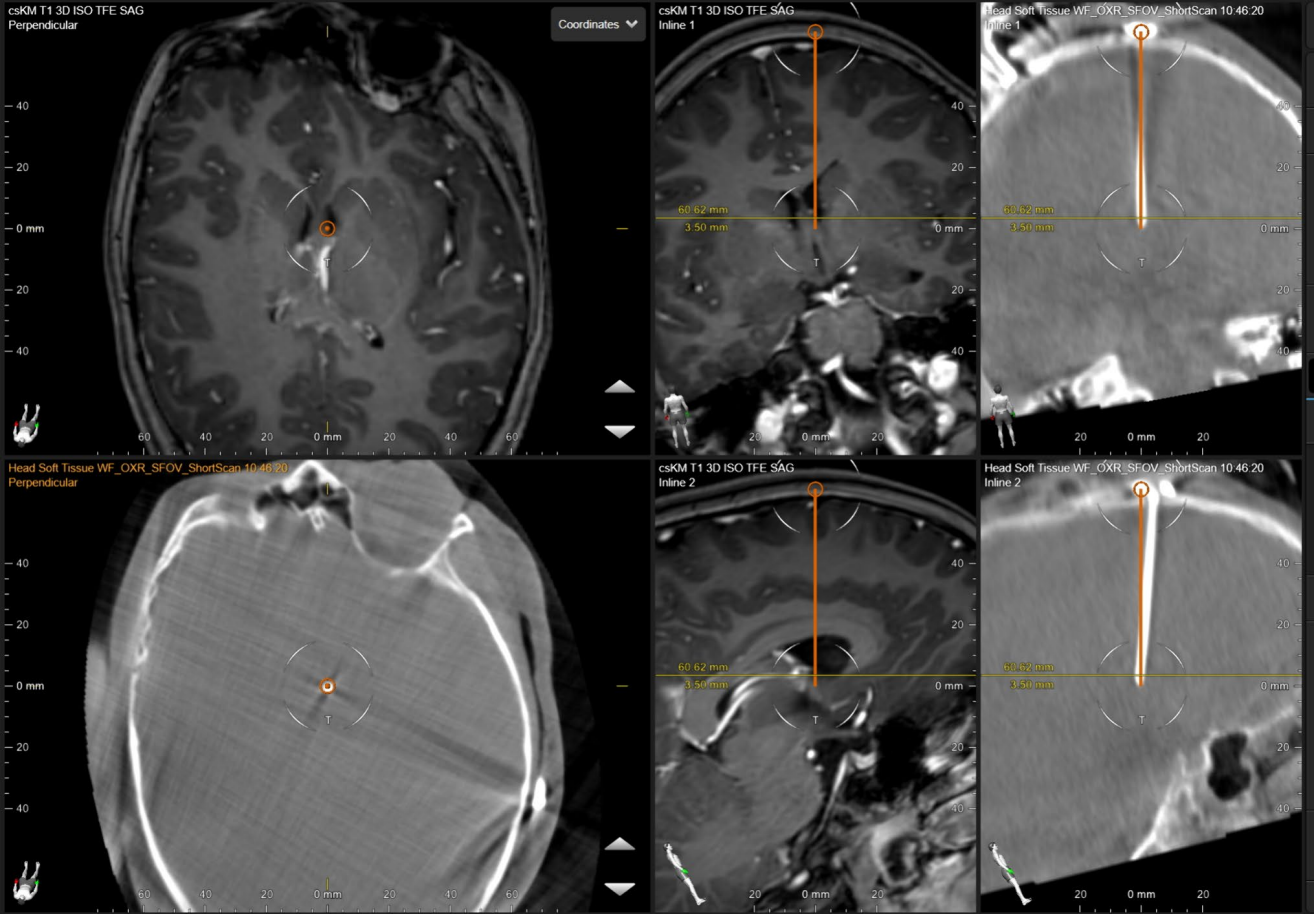
Intraoperative screenshot of the navigation system. The intraoperatively acquired images of the cone-beam-CT have been merged with the preoperative MRI within the neuronavigation system. The planned trajectory of the ventricular catheter is shown in orange

**Fig. 3:**
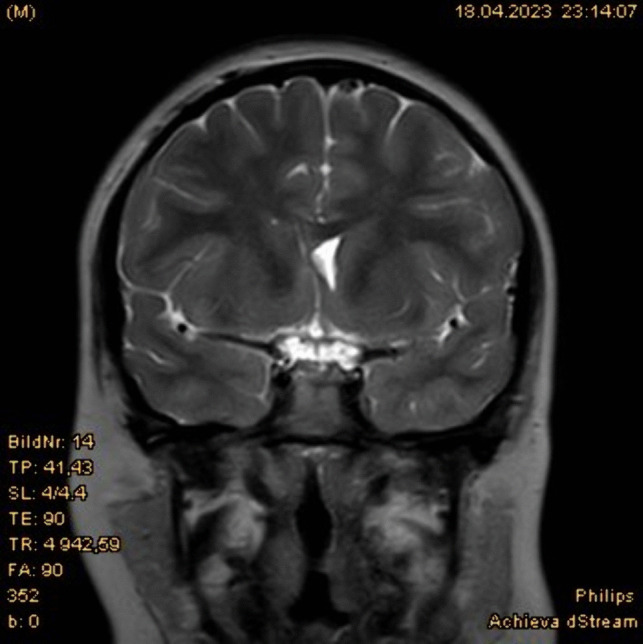
Postoperative MRI scan shows the tip of the catheter at the right frontal horn near the Foramen of Monroe and the reduced size of the right lateral ventricle

For placing the catheter, a Cushing canula was referenced into the BrainLab navigation system with the Instrument Calibration Matrix (ICM) and an Instrument Adapter Clamp S. The autopilot application was used to introduce the referenced Cushing canula into the right frontal horn. Cerebrospinal fluid was obtained for routine laboratory examinations. After removal of the Cushing canula, a standard ventricular catheter was inserted without stylet through the puncture canal. After verification of free CSF flow through the catheter, the shunt system with a Miethke proGAV2.0 valve system (Christoph Miethke GmbH, Potsdam, Germany) was connected and passed to the abdomen in standard fashion. After provisional closure of the wound and sterile drape coverage of the operative field, the Loop-X imaging unit was moved to the operating table. Lateral and anteroposterior localizing images were obtained before performing a circular scan. The overall radiation dose area product was 0.0003220306 Gy/m^2^. The automatic fusion of the acquired CT dataset with the preoperative MRI showed correct placement of the tip according to the preplanned trajectory (Fig. 2). Postoperative cranial MRI verified correct position also after mobilization of the patient. (Fig. 3). No periprocedural complications occurred.

## Conclusions

Intraoperative cone-beam CT can be used for registration, intraoperative neuronavigation, and verification of landmarks. Consequently, intraoperative CT allows the verification of catheter placements in selected patients with a high precision and at minimal radiation dosage in the pediatric population. The Loop-X imaging unit is a very fast and instant verification that may help to reduce revision procedures due to suboptimal ventricular catheter placement. However, the availability of the unit limits its use to few centers where it is available in hybrid operating theatres where it is used in a large variety of different indications. A larger cohort of patients will be analyzed in a prospective study to further verify the reliability of the technique.

## Data Availability

No datasets were generated or analysed during the current study.
